# Cost effectiveness of home care versus hospital care: a retrospective analysis

**DOI:** 10.1186/s12962-023-00424-0

**Published:** 2023-02-02

**Authors:** Iris Megido, Yael Sela, Keren Grinberg

**Affiliations:** 1 Operating Division, Maccabi Healthcare Services, Tel Aviv, Israel; 2 Department of Nursing Sciences, Faculty of Social and Community Science, Ruppin Academic Center, Emek- Hefer, Israel

## Abstract

**Background:**

Increased utilization of health services due to population growth affects the allocation of national resources and budgets. Hence, it is important for national policy. Home hospitalization is one of the solutions for dealing with the growing demand for hospital beds and reducing the duration of hospitalization and its costs. It is gradually becoming part of the regular care in many health systems, yet, studies on the economic aspects of Community-Based Home Hospitalization (CBHH) implementation in Israel are few. The aim of this study is to examine costs of CBHH in comparison to costs of inpatient hospital care in the Israeli public health system.

**Methods:**

Retrospective data was collected using document research in databases. A review of the costs of patients in CBHH at Maccabi Healthcare Services (MHS) was conducted. A total of 3374 patients were included in this study: 1687 patients who were in CBHH, and 1687 age- and sex-matched patients who were hospitalized in an internal department (the control group). The study population included the patients admitted to CBHH from January 2018 to July 2020, and patients admitted to internal medicine departments during the same period.

**Results:**

The number of hospitalizations during the follow up period were statistically significantly lower in the CBHH group compared with the control group (M = 1.18, SD = 0.56 vs. M = 1.61, SD = 1.29, p < 0.001). In addition, the mean number of hospitalization days was also statistically significantly lower for 4.3 (SD = 4.5) for CBHH patients compared to the control group (M = 4.3 days, SD = 4.5 vs. M = 7.5 days, SD = 10.3, p < 0.001). Furthermore, the mean cost per day was statistically significantly higher for inpatient hospitalization compared to CBHH (M = 1829.1, SD = 87.5 vs. M = 783.2, SD = 178.3, p < 0.001). Older patients, patients with diabetes and patients hospitalized in hospitals had a higher number of hospitalization days.

**Conclusions:**

The costs of CBHH seem to be lower than those of inpatient care. Managing CBHH is characterized by constantly measuring financial feasibility that would be an impetus for further development of this service.

## Introduction

Population growth, and specifically the growth in aging populations, the rise in the rate of chronic morbidity, and grave budgetary predicaments that led to a lack of resources and medical personnel, have formed a situation in which many public health systems must deal with the challenge of deficient resources versus the growing demand for services [11].

These challenges have not bypassed Israel’s public health system. The national expenditure on health in Israel is 7.3% of the gross domestic product (GDP), which is low compared to the average of 8.9% of the GDP in OECD countries [10]; National Expenditure on Health 1962–2018, 2019). The rate of physicians employed in Israel per 1000 people is lower than the OECD average. Also, the rate of nurses employed in Israel per 1000 people and the average hospital stay in Israel is lower than in OECD countries. This is compatible with the low rate of beds and high occupancy of hospital beds in Israel, which is second highest of all OECD countries [6], and indicative of maximal utilization of the bed infrastructure. The resulting situation is a significant overload in the inpatient and emergency rooms in Israel, which is grave and is not expected to improve. These combined challenges lead to the unequivocal and inevitable conclusion that the current model of medical practice does not provide a suitable response to these challenges and is not sustainable [4]. Therefore, a systemic change in the concept of hospitalization in Israel is essential due to the lack of hospital beds and the structure of Israel’s health system.

## Background

### Community-Based home hospitalization (CBHH)

Home hospitalization is one of the solutions for dealing with the growing demand for hospital beds, and reducing the duration of hospitalization and its costs. Home-based hospitalization has several definitions. These include: “hospitalisation or active treatment of a patient at home for a predefined period in order to shorten an existing hospital stay or to prevent it in defined facilitating conditions” [17], and “a service that provides in-home hospital care to patients with complex clinical conditions who would be hospitalized in conventional facilities due to an acute episode, and require 24/7 monitoring and follow-up that is only available in the hospital” [8, 23]. Community-based home hospitalisation services are gradually becoming part of the standard care in many healthcare systems in the form of different service models and for an extensive range of diagnoses.

Notably, home care that does not replace hospital care is not included under the definition of home hospitalisation. Home hospitalisation is intended for suitable patients of all ages, but in practice more than 90% of patients are elderly, mainly aged 75 and older, who are hospitalised often and normally for lengthier periods than average. Therefore, home hospitalisation has entered medical consciousness and practice as a therapeutic tool in the world of geriatric medicine [9].

Furthermore, hospitalisation is costly for health systems and is a health risk for many patients, including medical complications related to the hospital stay, such as infections that might be contracted during a hospital stay, falls, unnecessary tests, and mistaken medications, as well as potential cognitive and functional harm to patients, particularly the elderly, due to being in a strange place where they have no control over their fate [5, 33, 37]. In addition, high occupancy rate contributes to the development of antibiotic resistant infections in hospitals, which are estimated to cause the death of 4000–6000 patients every year The State Comptroller and Ombudsman of Israel [36]. This has led to a change in the location in which treatment is provided. Home hospitalization has also grown due to the development of home care equipment and technologies, the wish to let patients choose where they want to be treated, to cut costs and hospital days, and to improve patients' health outcomes [3, 5, 18]. The diversion of medical treatments from hospitals to the community and into patients’ homes, and the development of home hospitalization as an alternative to inpatient care, have been gradually developing in the Western world in the last two decades as part of the global healthcare trend aimed at coping with the shortage of resources, overload of the health system, reducing complications of hospital stays and improving the quality of care and patient wellbeing [28]. In the US, the “Hospital at Home Program” was developed by researchers at the Johns Hopkins Schools of Medicine and Public Health in Baltimore, Maryland, and it was first implemented in 1995. The main purpose was to create an alternative treatment option for elderly patients who either refused to go the hospital or were at higher risk of hospital-acquired infections and other adverse events. According to early trials of this program, the costs of at home care was 32% less than traditional hospital care and consequently [20, 22].

Since then, many countries have adopted and exploited the principles of the program and have developed similar services. For example, in Canada, the multifaceted evidence-based INSPIRED COPD Outreach Program (Implementing a Novel and Supportive Program of Individualized Care for Patients and Families Living with REspiratory Disease) was first implemented in Halifax, Nova Scotia in 2010. Multidisciplinary healthcare teams provide a holistic home care services to the patients and their families [27]. In the recent decades, several countries have begun to expand home hospitalization services with great success. For example,

Australia (in the state of Victoria) Spain and France. Other countries such as Canada and England have local initiatives. In the United States, home hospitalization was recognized for Medicare funding at the end of 2020 as part of the Corona emergency provisions and the service has been in an accelerated support process since then.[13–15, 17, 24, 35]. The implementation of home-based hospitalization optimized the use of resources by providing health services for specific groups who do not require conventional hospitalization [8]. In Israel, the average hospital stay is 5.2 days compared to an OECD average of 6.4. This is compatible with the low rate of beds and high occupancy of hospital beds in Israel (93.8%), which is second highest of all OECD countries–preceded only by Ireland [6], and indicative of maximal utilization of the bed infrastructure. The low bed ratio per capita is a result of a long-standing government policy aiming to transfer as many treatments as possible to the community, and to control hospitalisation costs [32]. In 2016, the bed occupancy rate in general hospital wards in Israel was 94%, versus an average of only 75% in European Union countries and in similar countries [10]. The resulting situation is a significant overload in the inpatient and emergency rooms in Israel.

In recent decades, Israeli health maintenance organizations (HMOs), established various services to care for the population in the community and at home, but a home hospital service–where a patient receives services at home instead of being admitted to a hospital–did not exist. The HMOs are, not-for-profit health maintenance organizations which provide universal coverage to all citizens and permanent residents of Israel with access to a statutory benefits package. Specifically, to home care in Israel–inpatient hospitalization and home care are both paid in full and have a budgetary coverage by the HMO (Each individual freely chooses from among four competing). Generally, Israel has a national health insurance system that provides. The healthcare system is financed by general taxes and an earmarked payroll tax (health tax). These funds are allocated to the health plans according to a capitation formula, with risk adjustment intended to sufficiently compensate the plans for the cost of members.

In 2018, Maccabi Healthcare Services (MHS), the second largest HMO in Israel, was the first to establish a community-based home hospitalization (CBHH) program, because it understood that the patient’s place was in the community, and following the wish to improve patient's well-being and their responsiveness to care. This CBHH program constitutes a breakthrough and a first-of-its-kind model in Israel, and it is expected to result in reducing hospital overload and costs, while providing best personal care to patients requiring hospitalisation. CBHH represents a dramatic change to the traditional concept of acute hospitalization, as well as an organizational change that must be managed within the Israeli health system.

Maintaining financial resource management and reducing health costs is a significant challenge and a necessary need for the continued existence of any health organization. According to Perroca and Ek [30], who reviewed home hospitalisation programs in Sweden, such programs have clinical and economic efficacy due to the need to reduce costs in the public sector, which are increasing with the rise in life expectancy and in the number of people over 80 in the country. Aimonino Ricauda et al. [1], conducted a comparative study in Torino, Italy with 104 patients with chronic obstructive pulmonary disease (52 in home hospitalisation and 52 in a control group). The results indicated a reduction in repeat hospitalisations among the group of patients in home hospitalisation during the 6 months after discharge, lower direct costs in home hospitalisation versus hospital stay, and higher satisfaction among patients in home hospitalisation [2].

A three-year comparative study between patients in home hospitalisation and a control group of patients in hospital care, conducted among 507 adult patients in New York showed that patients in home hospitalisation had shorter acute hospitalisation, less complications, less repeat hospitalisations, and lower costs than the control group [13, 14]. reported less hospital days and a considerable cut in costs among 1276 adult patients in home hospitalisation, especially among those aged 75 and older. Hernandez et al. [17] conducted a 10 year prospective evaluation to investigate the clinical outcomes, costs and barriers to embracing a home hospitalisation/early discharge program among 4165 patients with respiratory tract illnesses, as well as patients after surgery, cardiac, oncological, and others with acute illnesses The results showed high acceptance of the service (82%), very high satisfaction with the service by patients and carers (98.5% satisfied) and shortening the hospital stay by one day. Despite the rise in patient complexity over the years, the mortality rate remained identical (2%), the rate of repeat hospitalisations within 30 days diminished by 2% and the rate of visits to the emergency room diminished by 1%. With regard to costs, at first the results showed that home hospitalisation was cheaper, but at present, there is no difference in the payment schemes for both types of services [17]. Levine et al. [26] suggested, based on trials conducted using an at-home care delivery model, that the cost associated with acute care episode was 52% lower than those of traditional inpatient care [26].

While, in the long run, the introduction of new medical services and technologies has the power to reduce future health costs as shown in several studies that have examined the economic effects of chronic illness [12, 25], in the short term, the question of the economic viability of home hospitalization to the Israeli HMOs must be examined. This is because in health economy, often when health services that replace existing health services are added, even if the new service is cheaper, in fact we create a supply of health services/new technologies that cause patients to consume more health services. Therefore, in the short-term overall health expenditures are increased. The cost savings of the new service or technology are often seen in the long run because they reduce complications and improve the overall health of patients [7, 29].

There are many studies on home hospitalization in the Western world; however, there are limited studies about the cost compared to inpatient hospitalization. Specifically, in the State of Israel where CBHH is a new phenomenon, the subject has hardly been studied.

Thus, the objective of this study was to examine costs of CBHH in comparison to costs of inpatient hospital care in the Israeli public health system. The provision of an empirical assessment of costs enables understanding the economic feasibility of the service.

### Research question

What is the cost of CBHH compared to the cost of inpatient care in the Israeli public health system? Our hypothesis was that the cost of CBHH would be lower than the cost of inpatient care.

### Design

In order to examine and compare the total cost of CBHH to that of inpatient care, retrospective data was collected using document research in databases.

### Method

**Fig. 1:**
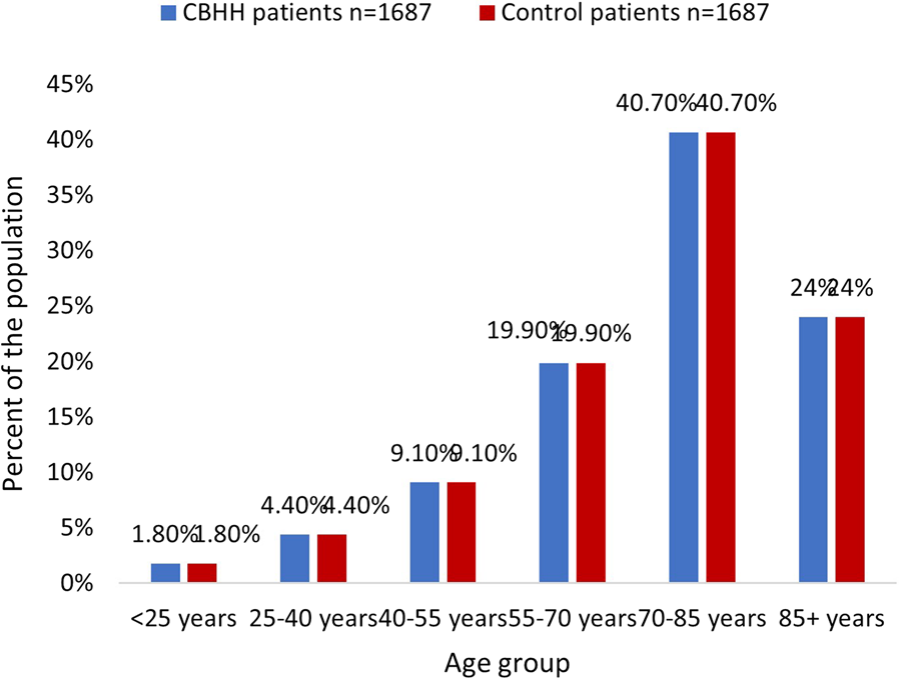
Distribution of the study population by age

**Fig. 2:**
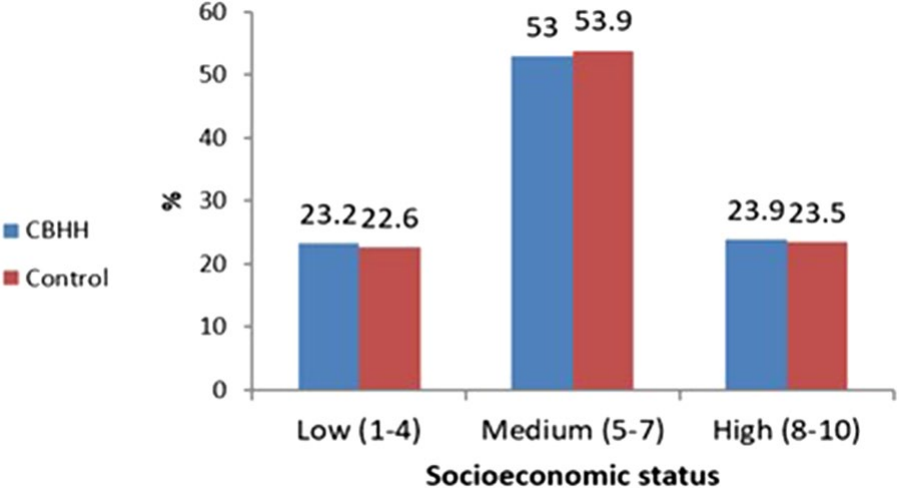
Distribution of the study population by socioeconomic status

**Fig. 3:**
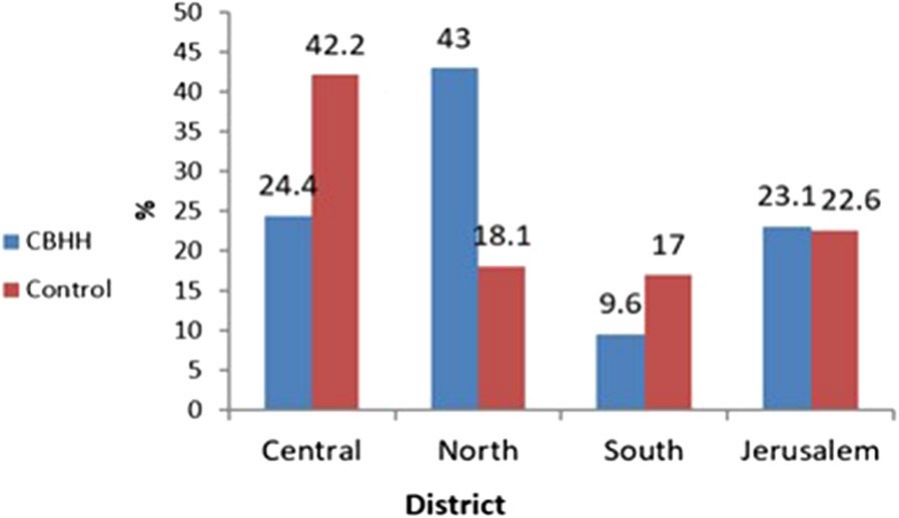
Distribution of the study population by place of residence

**Fig. 4:**
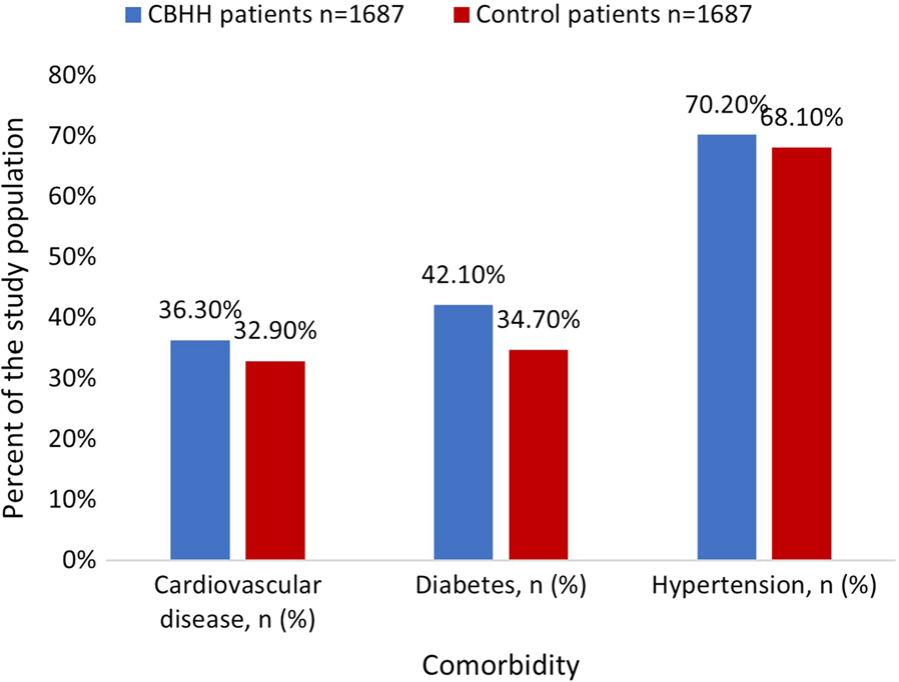
Distribution of the study population by comorbidities

A review of the costs of patients in CBHH at Maccabi Healthcare Services (MHS) was conducted. The study population included patients admitted to CBHH from January 2018 to July 2020, and patients admitted to internal medicine departments during the same period. Admission criteria require that the patient suffer from.

chronic diseases, such as cardiovascular diseases, Hypertension or Diabetes. Oncology patients or those who had undergone transplantations, were excluded from the analysis. The control group was matched 1:1 by sex and age to the CBHH population. A total of 3374 patients were included in this study: 1687 patients who were in CBHH, and 1687 age- and sex-matched patients who were hospitalized in an internal department (the control group). Baseline characteristics of CBHH patients vs. the control group were compared using chi-squared test for categorical variables and t-test for independent samples for continuous variable. No statistically significant differences in sociodemographic characteristics were found between the study groups. The descriptive statistics of the study population are presented in Table 1 and in Figs. 1, 2, 3, 4.

**Table 1:** Patient characteristics

Variable	CBHH patients n = 1687	Control patients n = 1687	Statistical test	P value
Sex, n (%)				
Men	868 (51.5%)	868 (51.5%)	χ^2^_=0_	1.0
Women	819 (48.5%)	819 (48.5%)		
Age (years), mean (SD)	72.3 (17.1)	72.3 (17.0)	t_(3372)_ = − 0.03	0.975
Age group, n (%)				
< 25 years	31 (1.8%)	31 (1.8%)		
25–40 years	74 (4.4%)	74 (4.4%)		
40–55 years	154 (9.1%)	154 (9.1%)	χ^2^ = 0	1.0
55–70 years	336 (19.9%)	336 (19.9%)		
70–85 years	687 (40.7%)	687 (40.7%)		
85 + years	405 (24%)	405 (24%)		
Socioeconomic status, n (%)				
Low (1–4)	390 (23.2%)	380 (22.6%)		
Medium (5–7)	892 (53%)	908 (53.9%)	χ^2^ = 0.32	0.853
High (8–10)	402 (23.9%)	396 (23.5%)		
Comorbidities				
Cardiovascular disease, n (%)	612 (36.3%)	555 (32.9%)	χ^2^ = 4.26	0.039
Diabetes, n (%)	710 (42.1%)	585 (34.7%)	χ^2^ = 19.58	< 0.001
Hypertension, n (%)	1184 (70.2%)	1149 (68.1%)	χ^2^ = 1.70	0.192

*SD* standard deviation

Data were collected from the MHS Business Intelligence (BI) system. The MHS database contains longitudinal data on a stable population of 2.3 million people insured by MHS. Data are automatically collected, and include comprehensive laboratory results from a single central lab, full pharmacy prescription and purchase data, physician visits, imaging, and extensive demographic information on each patient. The database includes several automatically formulated registries including diabetic, cardiovascular and hypertension registries.

### Analysis

Significant differences in baseline characteristics between the analysis groups (CBHH vs. control group) were determined using chi-squared test for categorical variables and t-test for independent samples for continuous variables. A two-sample T-test for independent samples was applied to test the statistical significance of the difference between the CBHH group and the control group in the dependent variables, hospitalization days, and cost. All tests were two-tailed, and a p-value of 5% or less was considered statistically significant. All statistical analyses were performed using SPSS version 25 (IBM Corporation Inc.).

## Results

In this study, the hypothesis was that CBHH would have lower cost compared to inpatient care. Table 2 describes the results of the comparison between CBHH patients and inpatient hospital patients.

**Table 2:** Comparison of hospitalizations and cost by groups

	CBHH patients n = 1687		Control patients n = 1687		t_(3372)_	P value
	mean	SD	mean	SD		
Number of hospitalizations	1.18	0.56	1.61	1.29	12.57	P < 0.001
Hospitalization days	4.3	4.5	7.5	10.3	11.62	P < 0.001
Cost per day (cost units)	783.2	178.3	1829.1	87.5	216.24	P < 0.001

*SD* standard deviation

P value by two-sample T-test for independent samples

The number of hospitalizations during the follow up period (January 2018-July 2020) were statistically significantly lower in the CBHH group compared to the control group (M = 1.18, SD = 0.56 vs. M = 1.61, SD = 1.29, p < 0.001).

The mean number of hospitalization days was also statistically significantly lower for 4.3 (SD = 4.5) for CBHH patients compared to the control group (M = 4.3 days, SD = 4.5 vs. M = 7.5 days, SD = 10.3, p < 0.001).

The mean cost per day was calculated by dividing the direct hospitalization cost by the number of hospitalization days (presented in cost units). The mean cost per day was statistically significantly higher for inpatient hospitalization compared to CBHH. (M = 1829.1, SD = 87.5 vs. M = 783.2, SD = 178.3, p < 0.001).

Hence, the hypothesis was confirmed.

Multiple linear regression performed to examine the relative effect of sex, age and comorbidities (heart disease, diabetes, hypertension) on hospitalization days, showed that age, diabetes and study group were significant, with an explained variance of R^2^ = 5.6% (p < 0.001). So older patients, patients with diabetes and patients hospitalized in hospitals had a higher number of hospitalization days (Table 3).

**Table 3:** Multiple linear regression for hospitalization days by sex, age, comorbidities, and study groups

Hospitalization days	B	SE B	Beta	P value
Group (CBHH = 1)	− 3.28	0.27	− 0.203	< 0.001
Sex (female = 1)	0.06	0.28	0.004	0.839
Age	0.04	0.01	0.081	< 0.001
Diabetes	1.10	0.29	0.066	< 0.001
Cardiovascular disease	0.57	0.31	0.033	0.070
Hypertension	0.19	0.36	0.011	0.592

In summary, the analysis showed that the costs of CBHH are significantly lower than those of inpatient hospitalization. The cost of CBHH is lower despite the fact that the CBHH group included more chronically ill patients than the control group, but nevertheless they did not utilize more days of hospitalization. This fact strengthens the hypothesis that the costs of CBHH are lower than those of inpatient hospitalization.

This finding is consistent with the literature discussing the fact that contemporary health care organizations face a shortage in many areas and require quality, low-cost alternatives to first-line appointments with medical service providers and inpatient wards [16].

## Discussion

The perception among stakeholder managers in the Israeli public health system is that aside from the health benefits of CBHH for patients, it is also very important to develop the service as one that may reduce the costs of hospitalization in the entire health system. HMOs have begun to develop CBHH services in the last three years under the assumption that they will be able to save hospitalization costs.

The findings of the current study show that the costs of CBHH are significantly lower than those of inpatient care, in the research group versus the control group. Moreover, although in the research group there were more chronically ill patients than in the control group, patients in CBHH still had less hospital days and their cost was lower.

This finding can be explained by the fact that chronic patients have a higher probability of complications in inpatient care due to their underlying health problems, which may lead to extended hospital hospitalization days and expenses for additional tests and treatments. These additional expenses are saved in the treatment of chronic patients with CBHH. Reinforcement to this finding is provided by the literature. Several studies have reported that CBHH costs were lower than the costs of inpatient care [13, 14, 34, 35]. Other studies reported ambivalent findings regarding the financial feasibility of CBHH. An analysis of studies conducted with thousands of patients, reported a decrease in the number of hospital days, but the answer to the question of whether CBHH affects overall costs was not unequivocal [15]. A 10 year meta-analysis reported a decrease in costs in the first years, but no difference in costs between CBHH and inpatient care in later years [17]. The ambivalence that emerges occasionally in the literature on this topic concerning the question of CBHH financial feasibility can be explained by the difficulty with assessing the overall costs of the service in the short-term compared to inpatient care, versus the anticipated savings in the long-term, as manifested in the forecast for reduced complications in inpatient care, such as mental harm and functional deterioration as well as cross-infections. It is possible that in home hospitalization interventions, some costs are transferred to patients and caregivers. The treatment of a family member who is hospitalized at home may require treatment for quite a few hours and a cost derived from loss of working days, electricity and food costs and is a real concern. Time allocations to care, if not provided by family caregivers, may have necessitated the acquisition of a privately funded caregiver. Measuring only health system costs, whether publicly or privately financed, may therefore lead to an inaccurate estimate of relative resource costs associated with alternative health care settings or interventions, particularly when family costs represent a large portion of overall costs. This is another reason for the problem in measuring the cost effectiveness of home hospitalization [19, 31]. Another view of the financial feasibility concerns ‘supply and demand’, where there is a concern among managers and policy makers that when the health system adds new health services, even if these are cheaper than the existing ones, their supply will increase consumption, and thus increase the overall health expenditure. Namely, the concern is that patients who before CBHH was established would have remained at home and received treatment in the community, they or their doctor would now prefer CBHH treatment. This aspect is related to managerial responsibility to control the service. Today’s management world places cost reduction in organizations as a major challenge for managers in all public organizations. In healthcare organizations, the need to reduce costs is at the heart of the management goals [21], when for more than two decades the WHO has been measuring national health systems according to criteria of efficiency and cost [38].

The discussion should be concluded with the understanding that even if there is no blanket agreement at present that CBHH services are necessarily financially feasible for all public health systems, many countries in the Western world are constantly developing additional innovative CBHH services, while expressing trust in the service and recognition of the need and of the many advantages, including long-term financial feasibility. The current study sheds new light regarding the financial feasibility of Home hospitalization (HH). As a substitute to in-patient care, this is an interest growing domain, particularly amongst the elderly, but there is still a debate about the general economic justification.

## Limitations

The study included relatively small sample size, its cross-sectional design, which only allows to view the results in a specific point in time. It is possible that the study population does not represent all the insured patients in Maccabi Health Services. Furthermore, much more clinically robust and detailed information on function, cognition, frailty, mental health, and medical complexity is required to have any confidence that the groups are comparable. It is possible that the study population does not represent all the insured in Maccabi Services and Health and in Israel in general. In addition, the calculation of the specific medical costs and medical dimensions that may affect the costs should also be examined in follow-up studies. This is a preliminary study and more studies should be done in order to claim that home-based care is more cost-effective than hospital-based care.

## Conclusion

The findings of this study promote the understanding that CBHH is an economically viable service to the healthcare system. The conclusion emerging from this discussion is that managing CBHH is characterized by constantly measuring financial feasibility that would be an impetus for further development of this service.

## Data Availability

The data that support the findings of this study are available from Maccabi Healthcare Services, but restrictions apply to the availability of these data, which were used under license for the current study, and so are not publicly available. Data are however available from the authors upon reasonable request and with permission of Maccabi Healthcare Services.

## Abbrevations

CBHH: Community-based home hospitalization
MHS: Maccabi healthcare services
HMO: Health maintenance organizations
